# Inhibitory Effect of *Phragmanthera Incana* (Schum.) Harvested from Cocoa (*Theobroma Cacao*) and Kolanut (*Cola Nitida*) Trees on Fe^2+^ induced Lipid Oxidative Stress in Some Rat Tissues - *In Vitro*

**Published:** 2015-03

**Authors:** O. T. Ogunmefun, T. R. Fasola, A. B. Saba, A. J. Akinyemi

**Affiliations:** 1 Department of Biological Sciences, College of Sciences, Afe Babalola University, Ado-Ekiti, Nigeria;; 2 Department of Botany, Faculty of Science, University of Ibadan, Ibadan, Nigeria;; 3 Department of Physiology, Biochemistry and Pharmacology, Faculty of Veterinary Medicine, University of Ibadan, Ibadan, Nigeria;; 4 Department of Biochemistry, College of Sciences, Afe Babalola University, Ado-Ekiti, Nigeria

**Keywords:** Phragmanthera incana, Cocoa, Kolanut, Antioxidant properties, Lipid peroxidation, Malondialdehyde

## Abstract

Evidence in both experimental and clinical studies has shown the participation of oxidative stress in the development and progression of diabetes mellitus. This study therefore, sought to investigate the inhibitory effect of methanolic extract of *Phragmanthera incana* leaves, a mistletoe species harvested from Cocoa (*Theobroma cacao*) and Kolanut (*Cola nitida*) on FeSO_4_ induced lipid peroxidation in rat pancreas, liver, kidney, heart and brain *in vitro*. The methanolic extract was prepared with 90% methanol (v/v); subsequently, the antioxidant properties and inhibitory effect of the extract on Fe^2+^ induced lipid peroxidation in some rat tissues were determined *in vitro*. Incubation of the different rat tissues homogenate in the presence of Fe caused a significant increase in the malondialdehyde (MDA) contents of the tissues. However, the methanolic extracts of *Phragmanthera incana* leaves harvested from both Cocoa and Kolanut trees caused a significant decrease in the MDA contents of all the tissues tested in a dose-dependent manner. However, the extract of *Phragmanthera incana* leaves harvested from kolanut trees had a better inhibitory effect on Fe^2+^- induced lipid peroxidation in the rat tissues homogenates than that of *Phragmanthera incana* leaves harvested from cocoa trees. This higher inhibitory effect could be attributed to its significantly higher antioxidant properties as typified by their phenolic content, DPPH radical scavenging ability and reducing power. Therefore, oxidative stress associated with diabetes and its other complications could be potentially managed/prevented by harnessing *Phragmanthera incana* leaves as cheap nutraceuticals. However, *Phragmanthera incana* leaves harvested from kolanut trees exhibited better antioxidant properties.

## INTRODUCTION

Diabetes mellitus (DM) is a chronic disease caused by inherited or acquired deficiency in insulin secretion and such a deficiency result in increased blood glucose level, which in turn can damage many of the body’s systems, including blood vessels and nerves (1). Increasing evidence in both experimental and clinical studies have shown that the role of oxidative stress in the development and progression of diabetes mellitus (2). This is usually accompanied by increased production of free radicals such as superoxide radicals, hydroxyl radicals etc or impaired antioxidant defenses (3). Free radicals are formed by increasing in diabetes by glucose oxidation, non-enzymatic glycation of proteins, and the subsequent oxidative degradation of glycated proteins. Abnormally high levels of free radicals and the simultaneous decline of antioxidant defense mechanisms can lead to damage of cellular organelles and enzymes, increased lipid peroxidation, and development of insulin resistance (3). It is well established, that free radicals are associated with process that lead to cell degeneration, especially in the brain, pancreas and other tissues (4).

High levels of both Cu and Fe, with low levels of Zn and Mn play a crucial role in brain cancer and in neurodegenerative disorders such as Lou Gehrig’s disease, Huntington’s disease and Alzheimer’s disease (5). Ferrous has been shown to cause oxidative damage by acting catalytically in the production of ROS which have the potential to damage cellular lipids, nucleic acids, proteins and carbohydrate resulting in wide ranging impairment in cellular function and integrity (6). ROS can directly attack the polyunsaturated fatty acids of the cell membranes and induce lipid peroxidation. Malondialdehyde (MDA) is the end-product of lipid peroxidation, which is a process where reactive oxygen species (ROS) degrade polyunsaturated fatty acids. This compound is a reactive aldehyde and is one of the many reactive electrophile species that cause toxic stress in cells and form advanced glycation end-products. The production of this aldehyde is used as a biomarker to measure the level of oxidative stress in an organism (7). However, consumption of foods rich in antioxidant phytochemicals may help fight degenerative diseases caused by oxidative stress by improving body’s antioxidant status by either scavenging or mopping off reactive Oxygen species (ROS).

The medicinal value of plants have assumed a more important dimension in the past few decades owing largely to the discovery that extracts from plants contain not only minerals and primary metabolites but also adverse array of secondary metabolites with antioxidant potential (8). The therapeutic effects of several plants and vegetables, which are used in traditional medicine, are usually attributed to their antioxidant compounds. Antioxidants are also used to preserve food quality mainly because they arrest oxidative deterioration of lipids. Plant-based antioxidants are now preferred to the synthetic ones because of safety concerns (8). These factors have inspired the widespread screening of plants for possible medicinal and antioxidant properties, the isolation and characterization of diverse phytochemicals and the development and utilization of antioxidants of natural origin (9, 10). A profile of the chemical composition of a plant together with knowledge of its antioxidant activity will give a fair estimate of its therapeutic potential (8).

These antioxidants are polyphenolic compounds, which are found in all plants and in all parts of the plants (tree bark, stalks, leaves, fruits, roots, flowers, pods and seeds (11).

Polyphenolic compounds are an important group of secondary metabolites, which are synthesized by plants because of plant adaptation to biotic and abiotic stress condition such as infection, water stress, and cold stress (12). In recent years, phenolic compounds have attracted the interest of researchers because of their antioxidants capacity; they can protect the human body from free radicals, which are formed due to normal natural metabolism in aerobic cells. The reduced free radicals’ activity of flavonoids and phenolics is principally based on the structural relationship between different parts of their chemical structure (13). Polyphenolics are common constituents of the human diet, present in most foods and beverages of plant origin. They are considered to contribute to the prevention of various degenerative diseases.

*Phragmanthera incana* (mistletoe) is a woody parasitic shrub, stems to 2m long; of secondary jungle and bush savanna areas; from Sierra Leone to West Cameroons and Fernando Po Island (in the gulf of Guinea that forms part of Equatorial Guinea) , and extending across the Congo basin to Zaire and Angola. The plant is very variable in form, common and widely distributed (14). Young parts and perianth more or less densely covered with brown hairs; berries red. The plant is very variable in the shape and size of the flowers and leaves. Mistletoe is used mainly in Europe as a treatment for cancer (15). While American mistletoe is toxic, European mistletoe is considered to have medicinal properties till today. The Drug Digests states that “for several diseases, European mistletoe has been used to treat a wide variety of physical and mental conditions. Currently, it is best known as an additional therapy with other drugs and or radiation for treating cancer”. Some HIV/AIDS Organizations (NGO’s) also claim that it can help restore immune systems (16). Away from superstitious beliefs, mistletoe has been used in medicine to prove much of its older frame as “all healer”. The white-berried mistletoe (*Viscum album*) has been documented as a traditional treatment for diabetes and high blood pressure. In Nigeria, the Hausa and Fulani tribes of Northern Nigeria use mistletoe in the treatment of cancers and inflammations. (17). The African mistletoe, *Loranthus bengwensis* L. (Loranthaceae), has been widely used in Nigeria folk medicine to treat diabetes mellitus (18), but there is limited information on its potential use in the management/prevention of this disease associated with oxidative stress. Hence, the objective of this study is to investigate the inhibitory effect of extractible phytochemicals of *Phragmanthera incana* from kolanut and cocoa trees on Fe^2+^ induced lipid peroxidation in some rat tissues *in vitro*.

## MATERIALS AND METHODS

### Collection and Identification of Plant Samples

Fresh samples of *Phragmanthera incana* (mistletoe)were collected in a forest at Alesan Obolode, Owo metropolis, Nigeria. Authentication of the mistletoe species was carried out at the Forestry Research Institute of Nigeria with Forest Herbarium Index 108925. A voucher specimen was submitted at the Department of Botany, University of Ibadan Herbarium.

### Chemicals and Reagents

Chemicals and reagents used were of analytical grade while the water was glass distilled.

### Methanolic Extract Preparation

The samples were washed under running water, air dried after which the dried samples were ground to powder and kept dry in an air-tight container. Cold extraction method with methanol for 72 hours at room temperature was used (19, 20). 500 g of powdered mistletoe samples harvested from Cocoa and Kolanut were extracted separately with one litre of methanol each after which concentration of the filtrates were done using rotary evaporator and the extracts were further concentrated on water bath at a low temperature of 40°C to remove all solvents.

### Lipid Peroxidation Assay

**Experimental Animals.** Albino rats weighing between 130 and 150 g were purchased from the Central Animal House, Department of Biochemistry, Afe Babalola University, Ado-Ekiti, Nigeria. The rats were allowed access to food and water *ad libitum*. The animals were used in accordance with the procedure approved by the Animal Ethics Committee of the University of Ibadan, Ibadan, Nigeria.

**Preparation of Tissue Homogenates.** Five organs (heart, liver, kidney, pancreas and brain) were harvested from rats for lipid peroxidation assay to maintain their freshness and were placed on ice and weighed. This tissue was subsequently homogenized in cold saline (1/10 w/v) with about 10 strokes at approximately 1200 rev/min in a Teflon glass homogenizer (Mexxcare, mc14 362, Aayu-shi Design Pvt. Ltd., India). The homogenate was centrifuged (KX3400C Kenxin Intl. Co. Hong Kong) for 10 minutes at 3000 × g to yield a pellet that was discarded, and a low-speed supernatant (SI), which was kept for lipid peroxidation assay (21).

**Lipid Peroxidation and Thiobarbibutric Acid Reactions.** The lipid peroxidation assay was carried out using the modified method of Ohkawa *et al.* 1979 (22). Briefly, 100 μl of the SI fraction was mixed with a reaction mixture containing 30 μl of 0.1 M pH 7.4 Tris-HCl buffer, extract (0-100 μl) and 30 μl of 250 μM freshly prepared FeSO_4_. The volume was made up to 300 μl by water before incubation at 37°C for 2 hours. The colour reaction was developed by adding 300 μl 8.1% SDS (Sodium dodecyl sulphate) to the reaction mixture containing SI, this was subsequently followed by the addition of 600 μl of acetic acid/HCl (pH 3.4) mixture and 600 μl of 0.8% Thiobarbituric acid (TBA). This mixture was incubated at 100°C for 1 hour. Thiobarbituric acid reactive species (TBARS) produced were measured at 532 nm and expressed as Malondialdehyde (MDA) produced (% control) using Malondialdehyde standard curve (0-0.035 mM).

**Determination of Total Phenolic Content.** The total phenolic content was determined on the extracts using the method reported by Singleton *et al*. 1999 (23). Appropriate dilutions of the extracts were oxidized with 2.5 ml of 10% Folin-Ciocalteau’s reagent (v/v) and neutralized by 2.0 ml of 7.5% Sodium carbonate. The reaction mixture was incubated for 40 minutes at 45°C and the absorbance was measured at 765 nm in the spectrophotometer. The total phenolic content was subsequently calculated as gallic acid equivalent using gallic acid standard curve.

**DPPH Free Radical Scavenging Ability.** The free radical scavenging ability of the extracts against DPPH (1, 1-diphenyl-2 picrylhydrazyl) free radical was evaluated as described by Gyamfi *et al.*1999 (24). Briefly, an appropriate dilution of the extracts (1 ml) was mixed with 1ml of 0.4 mM methanolic solution containing DPPH radicals, the mixture was left in the dark for 30 min and the absorbance was measured at 516 nm. The control was carried out using 2ml DPPH solution without the test samples. The DPPH free radical scavenging ability was subsequently calculated.

DPPH scavenging ability (%) = [(Absorbance of Control − Absorbance of Samples)/Absorbance of Control] × 100.

**Determination of Reducing Property.** The reducing property of the extracts was determined by assessing the ability of the extract to reduce FeCl_3_ solution as described by Oyaizu, 1986 (25). A 2.5 ml aliquot was mixed with 2.5 ml of 200 mM Sodium phosphate buffer (pH 6.6) and 2.5 ml of 1% Potassium ferricyanide. The mixture was incubated at 50°C for 20 min and then 2.5 ml of 10% Trichloroacetic acid was added. This mixture was centrifuged at 650 rpm for 10 min. 5 ml of the supernatant was mixed with an equal volume of water and 1 ml of 0.1% Ferric chloride. The absorbance was measured at 700 nm. The ferric reducing antioxidant property was subsequently calculated using ascorbic acid as standard.

## RESULTS AND DISCUSSION

**Figure 1:**
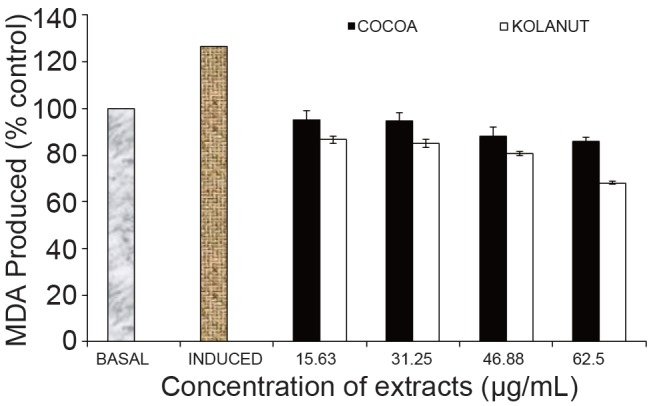
Effect of methanolic extract of Phragmanthera incana harvested from Cocoa and Kolanut trees on malondialdehyde level in rat kidney. MDA, Malondialdehyde; Basal, lipid peroxidation without Fe^2+^ as pro-oxidants and no extracts; Induced, lipid peroxidation with Fe^2+^ as prooxidants and no extracts.

**Figure 2:**
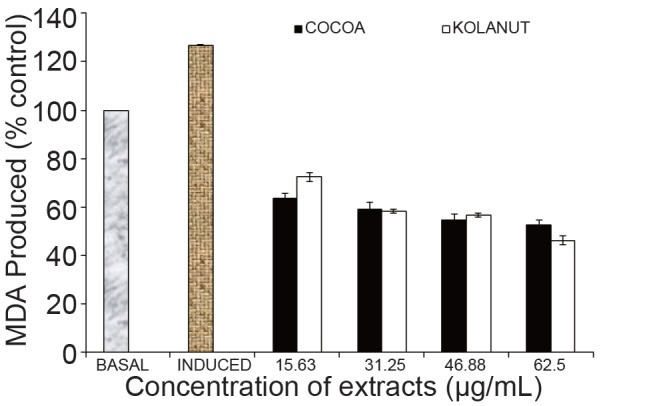
Effect of methanolic extract of P hragmanthera incana harvested from Cocoa and Kolanut on malondialdehyde in rat heart. MDA, Malondialdehyde; Basal, lipid peroxidation without Fe^2+^ as pro-oxidants and no extracts; Induced, lipid peroxidation with Fe^2+^ as prooxidants and no extracts.

**Figure 3:**
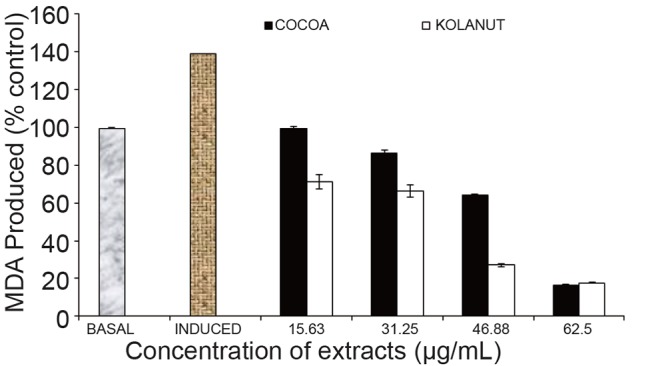
Effect of methanolic extract of Phragmanthera incana harvested from Cocoa and Kolanut trees on malondialdehyde in rat liver. MDA, Malondialdehyde; Basal, lipid peroxidation without Fe^2+^ as pro-oxidants and no extracts. Induced, lipid peroxidation with Fe^2+^ as prooxidants and no extracts.

**Figure 4:**
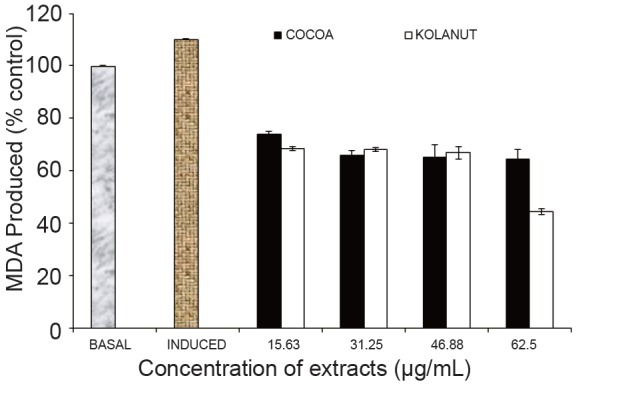
Effect of methanolic extract of Phragmanthera incana harvested from Cocoa and Kolanut trees on malondialdehyde in rat pancreas. MDA, Malondialdehyde; Basal, lipid peroxidation without Fe^2+^ as pro-oxidants and no extracts; Induced, lipid peroxidation with Fe^2+^ as prooxidants and no extracts.

**Figure 5:**
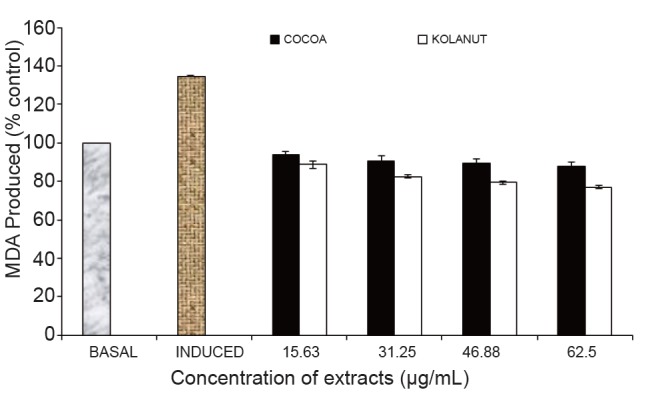
Effect of methanolic extract of Phragmanthera incana harvested from Cocoa and Kolanut trees on malondialdehyde in rat brain. MDA, Malondialdehyde; Basal, lipid peroxidation without Fe^2+^ as pro-oxidants and no extracts; Induced, lipid peroxidation with Fe^2+^ as prooxidants and no extracts.

Antioxidants are polyphenolic compounds found in all plant parts (leaves, stalks, fruits, pods, seeds, flowers, tree barks and roots) (Osawa *et al*., 1995) (11). This study established the fact that Fe^2+^ can induce lipid peroxidation in various rat tissues (kidney, liver, heart, pancreas and brain) as seen in Figures 1-5. The finding that Fe^2+^ caused a significant increase in the MDA content of the different tissues agreed with earlier report where Fe^2+^ was shown to be a potent initiator of lipid peroxidation in the brain (pro-oxidant) (26). The increased lipid peroxidation in the presence of Fe^2+^ could be attributed to the fact that Fe^2+^ can catalyze one-electron transfer reactions that generate reactive oxygen species, such as the reactive OH , which is formed from H_2_O_2_ through the Fenton reaction. Iron also decomposes lipid peroxides, thus generating peroxyl and alkoxyl radicals, which favors the propagation of lipid oxidation (27). Elevated Fe^2+^ content in the tissues had been linked to a host of degenerative diseases (28). However, *Phragmanthera incana* (mistletoe) harvested from both Cocoa (*Theobroma cacao*) and Kolanut (*Cola nitida*) trees inhibited MDA produced in rat kidney when compared to the induced (Fig. 1). It was observed that the mistletoe species harvested from Kolanut lowered the level of malondialdehyde (MDA) dose-dependently having the greatest effect at the highest concentration (62.5 µg/mL). The mistletoe from Cocoa also showed similar activity by lowering the percentage MDA in the rat kidney dose-dependently but not as effective as the mistletoe from Kolanut (Fig. 1).

Furthermore, the effect of the of the mistletoe extract from both plants on MDA produced in rats’ heart as a result of the induction by Fe^2+^ is shown in Fig. 2. At the lowest concentration of the extracts (15.63 µg/mL), mistletoe harvested from Cocoa showed a better activity in lowering the percentage MDA produced in the rats’ heart. The effects of the extracts from Cocoa and Kolanut at concentrations of 31.25 µg/mL and 46.88 µg/mL showed little or an insignificant difference in their ability to lower MDA i.e. their antioxidant activities; but at the highest concentration of the extracts (62.5 µg/mL), the mistletoe from Kolanut showed a better antioxidant property in lowering MDA in the rats’ heart (Fig. 2).

*Phragmanthera incana* harvested from Kolanut also manifested a better effect in lowering MDA in the rats’ liver dose-dependently. The best effect was seen at the 46.88 µg/mL concentration. At the highest concentration (62.5 µg/mL), the mistletoe from both plants were only slightly different from each other in their activities in lowering the MDA produced in the rats’ liver (Fig. 3).

The mistletoe extract from Kolanut at the highest concentration (62.5 µg/mL) showed its best antioxidant property in lowering MDA in rats’ pancrease. It also lowered MDA at other lower concentrations (15.53 µg/mL, 31.25 µg/mL and 46.88 µglmL) like the mistletoe from Cocoa; but the mistletoe from both plants were only slightly different in their activities at 31.25 µg/mL and 46.88 µg/mL concentrations (Fig. 4).

In addition, the effect of *Phragmanthera incana* harvested from both Cocoa and Kolanut followed similar pattern in lowering MDA in rats’ brain. The effect showed that both mistletoe inhibited MDA production dose-dependently with the mistletoe from Kolanut manifesting better inhibitory activity compared to the mistletoe from Cocoa (Fig. 5).

The results of this study is in agreement with other studies reported by Oboh * et al*., 2012 (29) for two ginger varieties and Akomolafe *et al.*, 2012 (30) for *Moringa oleifera* and *Newbuoldia laevis* leaves. The ability of this mistletoe extracts to inhibit the production of MDA in the various tissues could be as a result of their phytochemicals present which have antioxidant properties.

In an attempt to explain some possible mechanism through which the extracts prevent tissue damage against Fe^2+^ induced lipid peroxidation, some antioxidant propertiess (parameters) were determined.

**Figure 6:**
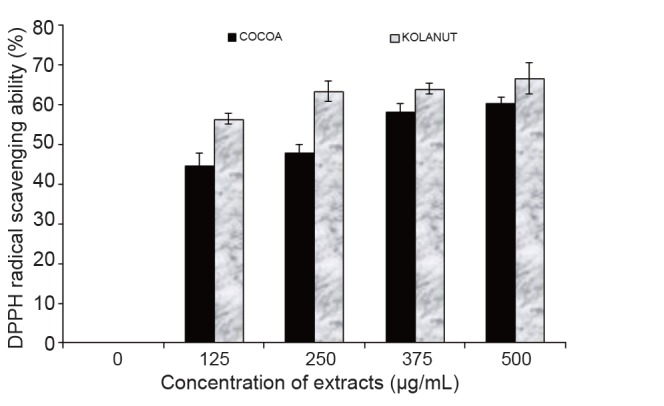
DPPH radical scavenging ability of methanolic extract of Phragmanthera incana harvested from Cocoa and Kolanut trees.

The results of the phenolic content and the ferric reducing antioxidant power (FRAP) as presented in Table 1 revealed that *Phragmanthera incana* (mistletoe) harvested from Kolanut had higher phenolic content and greater ferric reducing antioxidant power compared to the *Phragmanthera incana* harvested from Cocoa. The higher antioxidant activity of the mistletoe harvested from Kolanut was further supported by the result of its DPPH radical scavenging ability to be a better antioxidant agent than the mistletoe harvested from Cocoa (Fig. 6).

**Table 1:** The table of total phenolic content and ferric reducing antioxidant power of phragmanthera incana harvested from cocoa and kolanut trees

	Total Phenol	FRAP

*Phragmanthera incana* (mistletoe) harvested from host plants	Mean ± STD	Mean ± STD
Cocoa (*Theobroma cacao*)	5.566667 ±0.340783	1.318 ± 0.06364
Kolanut (*Cola nitida*)	7.55 ± 0.554256	3.07 ± 0.280014

Earlier studies had established a correlation between the phenol content of plant food and their antioxidant properties (31). The antioxidant activity of phenolics is mainly because of their redox properties, which allow them to act as reducing agents, hydrogen donors, free radical scavenger, singlet oxygen quenchers, and metal chelators (32, 33). Both the purported antioxidant activities and the reactive oxygen species scavenging properties of mistletoe *Phragmanthera incana* harvested from both Cocoa and Kolanut trees antioxidant phytochemicals are likely to explain, at least in part, their protective and rescuing abilities against degenerative diseases.

## CONCLUSSION

The mistletoe *Phragmanthera incana* harvested from both Cocoa and Kolanut trees inhibited Fe^2+^ induced lipid peroxidation in rat tissues *in vitro*. However, part of the mechanisms through which the extracts in *Phragmanthera incana* protect the organs may be through their antioxidant activities. However, *Phragmanthera incana* leaves harvested from kolanut trees exhibited better antioxidant properties. Oxidative stress associated with diabetes and its other complications could therefore be potentially managed/prevented by harnessing *Phragmanthera incana* leaves as cheap nutraceuticals. This finding therefore justifies the reason why it is being used ethnomedicinally to treat diverse array of disease conditions and ailments like diabetes, hypertension, inflammation, insomnia, cancer etc in different communities of the world which justifies the reason why mistletoe generally is termed an “all healer” plant.
